# Investigating Death Anxiety, Resilience, and Job Burnout Among Prehospital Emergency Personnel: A Multicenter Cross‐Sectional Study in Iran

**DOI:** 10.1002/hsr2.70751

**Published:** 2025-04-28

**Authors:** Ali Ghahramanipirsalami, Farzaneh Modaresi, Parisa Sabetsarvestani, Azizallah Dehghan, Saeed Abedi, Mostafa Bijani

**Affiliations:** ^1^ Student Research Committee Fasa University of Medical Sciences Fasa Iran; ^2^ Noncommunicable Diseases Research Center (NCDRC) Fasa University of Medical Sciences Fasa Iran; ^3^ Department of Medical Surgical Nursing, School of Nursing Fasa University of Medical Sciences Fasa Iran

**Keywords:** burnout, death anxiety, emergency medical services, mental health, resilience

## Abstract

**Background and Aims:**

As first responders to accidents and disasters, prehospital emergency personnel encounter psychological crises and mental stressors like death anxiety and job burnout. The present study aimed to investigate death anxiety, resilience, and job burnout in prehospital emergency personnel.

**Methods:**

This investigation employed a descriptive, cross‐sectional, observational, and multi‐centric study design. The research population encompassed prehospital emergency personnel affiliated with three universities of medical sciences in Fars Province, Southern Iran. A convenience sampling technique yielded a participant pool of 417 prehospital emergency personnel. Data was gathered using the Templer Death Anxiety Scale, Prehospital Emergency Personnel Resilience Scale and Maslach Burnout Inventory. Data were analyzed in SPSS‐26 through descriptive statistics, one‐way ANOVA, Pearson coefficient, and multiple linear regression. The significance level was set at *p* < 0.05.

**Results:**

The mean score for job burnout was 61.36 (SD ± 36.76), while the mean score for death anxiety was 99.5 (SD ± 94.10). These scores can be interpreted as indicative of high levels of both job burnout and death anxiety. Conversely, the mean resilience score of 96.37 (SD ± 77.06) suggests moderate resilience within the sample population. The findings revealed an inverse correlation between resilience and job burnout (*r* = −0.49, *p* < 0.001) and an inverse correlation between resilience and death anxiety (*r* = −0.51 *p* < 0.001). Also, the findings revealed a statistically significant positive correlation was found between job burnout and death anxiety (*r* = 0.37, *p* < 0.001). The results showed that age, work experience, resilience, and job burnout explained 44.4% of the variance in death anxiety (*R* ^2^ = 0.44, *p* < 0.001). In addition, work experience (*β* = 0.097, *p* = 0.043), resilience (*β* = 0. 208 *p* < 0.001), and job burnout (*β* = 0.337, *p* < 0.001) had the highest predictive impact on death anxiety.

**Conclusion:**

While the present study revealed high levels of death anxiety and job burnout among prehospital personnel, it also found their resilience to be moderate. Senior managers in prehospital emergency services should prioritize strategies to improve cognitive abilities, particularly resilience. By doing so, they can not only enhance employee well‐being but also potentially reduce job burnout and death anxiety.

## 1 Introduction

Prehospital emergency care functions as a community‐based health management system within the broader healthcare system, exhibiting a coordinated effort with the entire system itself [1]. Emergency medical services (EMS) are universally recognized as one of the most critical pillars in delivering medical care [2]. Prehospital emergency workers, under their role as first responders at accident, incident, and disaster scenes, consistently confront high levels of job anxiety and a multitude of challenges encompassing human resources, structural issues, management deficiencies, motivational concerns, equipment limitations, and inadequate support [1]. Conversely, the limited timeframe for intervention, the critical nature of patients' conditions, the expectations of patients' companions, the inherent dangers of the work environment, the fear of failing to save critically ill patients, the constraints on decision‐making in critical situations, and various human factors all contribute to the development of stress and death anxiety within this workforce [3]. Building upon Belexi's conceptualization, death anxiety can be understood as a constellation of abnormal thoughts, anxieties, and emotions about the terminal event–one's mortality, the apprehension surrounding the process of dying, and the concern for the demise of significant others [4]. Death anxiety is a multidimensional concept with emotional, cognitive, and experimental features and is defined as the feeling of anxiety or fear regarding the thought of death [5]. Death anxiety is the most crucial concern of human life and the core of all anxiety disorders. It is considered one of the most important factors in mental health [6]. According to the Terror Management Theory (TMT), it is posited that diverse factors—including cultural and social contexts, personal beliefs, value systems, and individual attitudes—substantially influence death anxiety. Consequently, investigations that examine death anxiety across varied cultural contexts significantly advance our conceptual understanding of this construct [7]. The EMS is a cornerstone within the healthcare system, operating around the clock to provide urgent and specialized medical attention to individuals confronting acute and life‐threatening medical issues. In these settings, healthcare professionals often confront death anxiety as they work tirelessly to save lives [8]. Despite their best efforts, not all patients survive, and EMS unit staff must cope with the emotional impact of death, leading to feelings of grief, guilt, and burnout. The fast‐paced and high‐pressure environment of EMS units can make it challenging for staff to process their emotions surrounding death [9]. The findings of the study by Asadi et al. (2021), showed that 46.7% of emergency medical technicians (EMTs) experienced severe death anxiety. Therefore, it is suggested that EMTs be continuously taught effective methods to deal with death anxiety and reduce the physical and mental disorders caused by this problem [10]. One prominent approach to addressing anxiety and its associated challenges lies in the concept of resilience. Resilience can be operationalized as an individual's capacity to adapt to stressful events, encompassing trauma, threats, interpersonal and familial discord, financial strain, and health concerns, thereby attenuating their negative ramifications [11]. Professional resilience transcends the absence of burnout; it necessitates positive adaptation and the expansion of an individual's internal resources [12].

Intriguingly, stress encountered within emergency work environments does not inevitably yield negative consequences. In some instances, it can even foster positive outcomes. These positive outcomes can be attributed to the inherent satisfaction derived from aiding others, imbuing individual challenges and efforts with meaning, fostering the realization of one's potential, and the gratification associated with fulfilling one's job duties and responsibilities competently [13]. Indeed, a study conducted by Pietrantoni and Prati yielded compelling evidence, demonstrating a significant level of resilience and positive personal transformations among first responders following exposure to critical incidents and stressful conditions [14].

Prehospital emergency medical teams operate within a highly dynamic environment, routinely responding to crises and accidents. However, this vital service comes at a cost, as personnel are susceptible to developing posttraumatic stress disorder, fatigue, and job burnout [15]. Job burnout, a concept initially introduced into the realm of clinical psychology research by Freudenberger in the 1970s, manifests as a syndrome characterized by physical‐emotional exhaustion [16]. This syndrome encompasses three distinct components: emotional exhaustion, depersonalization, and a diminished sense of personal accomplishment [17]. Job burnout is an internal, subjective phenomenon that fosters negative emotions and attitudes toward one's work, including dissatisfaction, exhaustion, and a waning sense of commitment [18]. Portela et al. (2015) studied job burnout syndrome in prehospital emergency personnel. Their findings were particularly alarming, with a staggering 80% of personnel exhibiting symptoms of burnout [19].

### 1.1 Background in Iran

The EMS department was first established in Tehran, Iran, in 1975, marking the beginning of EMS in the country. Following that, EMS started to develop in other cities gradually. There are currently 3300 EMS centers in the country. Personnel in EMS typically hold an associate or bachelor's degree in the field. Additionally, nurses with bachelor's or master's degrees may work in EMS. Every shift must have at least two staff members present. Further, EMS systems employ only males, and no female personnel are hired.

### 1.2 Research Gap and Significance of the Study

Given their pivotal role at the forefront of medical service delivery, prehospital emergency personnel are frequently the first point of contact for patients in distressful situations. The interplay between a lack of resilience and the repetitive nature of these encounters can culminate in job burnout, a subsequent decline in the quality of care provided, and potentially, harm to both the personnel and the patients they serve. Although previous research has individually explored resilience, occupational depression, and death anxiety among prehospital emergency personnel, no studies have specifically examined the interrelationships among these constructs within this population. This study focuses on the interplay between death anxiety, resilience, and job burnout among prehospital emergency personnel. It investigates how resilience can buffer the impact of death anxiety on professional performance and personal well‐being. The study also explores the potential for resilience‐building interventions to enhance prehospital emergency personnel's ability to cope with their work's emotional demands, contributing to improved mental health outcomes and patient care quality in prehospital emergency settings. By exploring the relationship between resilience and death anxiety levels among prehospital emergency personnel, this study fills a gap in the literature. It highlights the importance of further research in this area. It represents a crucial step towards recognizing and addressing the mental health challenges prehospital emergency personnel face, promoting their long‐term well‐being and effectiveness in their roles as frontline healthcare providers. Accordingly, the present study was conducted to investigate death anxiety, resilience, and job burnout in prehospital emergency personnel in southern Iran.

### 1.3 Research Questions

1. What are the death anxiety, resilience, and job burnout levels among prehospital emergency personnel?
2. Is there a statistically significant relationship between death anxiety, resilience, and job burnout levels among prehospital emergency personnel?
3. Is there a statistically significant relationship between death anxiety, resilience, and job burnout levels and demographic characteristics among prehospital emergency personnel?
4. What are the predictors of death anxiety based on the regression model?

## 2 Methods

The present study is a cross‐sectional observational multicenter research conducted between May to September December 2023 based on the strengthening of the reporting of observational studies in epidemiology statement (STROBE), a checklist for observational research [20].

### 2.1 Sample Size and Sampling Method

The research population comprised prehospital emergency personnel from three universities of medical sciences situated within Fars Province, Southern Iran. A convenience sampling approach was utilized to recruit participants, with a final sample size of 417 prehospital emergency personnel. In Iran, all prehospital emergency workers study at Iranian universities based on a common educational curriculum. Therefore, in terms of educational level, almost all were the same. In addition, the Iran EMS organization is responsible for managing this system, and the working and management conditions are almost the same throughout Iran. Therefore, the participants in this study could largely represent EMS personnel in Iran.

To ensure participant eligibility, a set of inclusion criteria were established. First, all participants were required to possess a minimum of 1 year of work experience in prehospital emergency care. Second, a demonstrable willingness to participate in the study was mandatory. Lastly, does not have previously known mental and physical disorders. The sole exclusion criterion was the participant's unwillingness to continue participation in the study for any reason. A convenience sampling approach was utilized for participant recruitment. The minimum sample size was determined based on the findings of a prior study by Almutairi et al. [17] and a pre‐established formula. This formula incorporates the confidence level (set at 95% for this study), the acceptable degree of difference (set at 5%), and a population standard deviation (assumed to be *σ* = 5). Employing this formula, the minimum required sample size was calculated to be 276. To enhance the generalizability and credibility of the study findings, the researchers opted to enroll a larger sample size of 417 participants.

### 2.2 Data Collection

After obtaining the required permissions to conduct the study, data gathering began with explaining the research goal to the prehospital emergency personnel under study and ensuring that their answers would remain private and anonymous. The researcher interviewed each participant individually and face to face once to collect the needed data using the study tools. The researcher met with the participants in a quiet area for 20–30 min during the break and between shifts.

### 2.3 Evaluation Tools

The data collection process employed a battery of standardized instruments. The first instrument was a demographic information questionnaire designed to gather information on participant characteristics relevant to the study. The second instrument was the Templer Death Anxiety Scale, a validated tool used to assess an individual's level of death anxiety. The third instrument was the Ebadi et al. Prehospital Emergency Personnel Resilience Scale, a measure specifically developed to evaluate resilience within this population. Lastly, the Maslach Burnout Inventory was utilized to assess participants' levels of job burnout.

The demographic information questionnaire included questions about age, work experience, education level, and marital status.

### 2.4 Death Anxiety Scale (DAS)

The Templer DAS is a widely used, self‐administered instrument designed to assess an individual's level of death anxiety. This 15‐item true/false scale can be further categorized into five subscales: Fear of Death (Items 1, 12, and 14), Fear of Pain and Illness (Items 2, 4, 6, and 13), Thoughts About Death (Items 5, 9, and 11), Time Passing and Life Being Short (Items 3, 7, and 10), and Fear of the Unknown (Items 8 and 15). Respondents agree with each statement using a dichotomous yes [1] or no (0) response format. However, scoring is reversed for Items 10, 11, 12, 13, 14, and 15 to account for the item's directionality. Total scores on the DAS range from 0 to 15, with higher scores reflecting greater levels of death anxiety. The psychometric properties of the DAS have been well‐established. The original study by Templer (1970) reported a Cronbach's alpha coefficient of 0.83, indicating good internal consistency [21]. Furthermore, the scale has demonstrated good reliability within Iranian samples, with a Cronbach's alpha coefficient of 0.91 reported by Sharif Nia et al. [22].

### 2.5 Prehospital Emergency Personnel Resilience Scale

Ebadi et al. (2019), Prehospital Emergency Personnel Resilience Scale is a standardized instrument specifically designed to evaluate resilience within this population. The questionnaire comprises 31 items, categorized across six distinct dimensions: Job Motivation (13 items), Communication Challenges (3 items), Social Support (2 items), Maintaining Calm (5 items), Self‐Management (5 items), and Stress Outcomes (3 items). Utilizing a 5‐point Likert scale (ranging from “Strongly Disagree” [1] to “Strongly Agree” [5]), the questionnaire yields a total possible score between 31 and 155. Within this instrument, higher scores correspond to greater levels of resilience. The scale's internal consistency has been demonstrated, with Ebadi et al. reporting a Cronbach's alpha coefficient of 0.91 in their initial study [23].

### 2.6 The Maslach Burnout Inventory (MBI) (1981)

The MBI is a well‐validated, 22‐item questionnaire that operationalizes burnout across three key dimensions: emotional exhaustion, depersonalization, and personal accomplishment. The MBI enjoys widespread use in various research and clinical settings due to its robust psychometric properties. Scores on the emotional exhaustion and depersonalization subscales are directly interpreted, with higher scores indicating greater burnout. Conversely, a reversal coding approach is employed during statistical analysis for the personal accomplishment subscale. To assess the prevalence of occupational burnout within a sample, researchers often utilize the quartile method, a well‐established statistical technique. Cut‐off scores for each dimension have been established to categorize the level of burnout experienced by participants. In the emotional exhaustion dimension, scores are categorized as follows: ≤ 16 (Low), 17–23 (Medium), and ≥ 24 (High). Similarly, depersonalization scores are categorized as ≤ 8 (Low), 9–11 (Medium), and ≥ 12 (High). The personal accomplishment subscale is categorized as ≤ 4 (Low), 5–22 (Medium), and ≥ 23 (High). Finally, an overall burnout level is determined by summing scores across all subscales, with categories established as ≤ 35 (Low), 36–53 (Medium), and ≥ 54 (High), reflecting the intensity of burnout experienced. The instrument's internal consistency has been extensively evaluated. Maslach and Jackson (1993) employed Cronbach's alpha, a reliability coefficient, and obtained excellent internal consistency for the emotional exhaustion subscale (*α* = 0.90) and strong internal consistency for both the depersonalization (*α* = 0.79) and personal accomplishment (*α* = 0.71) subscales [24]. Furthermore, the MBI's reliability within the Iranian context has been confirmed by Akbari et al. (2010), with a Cronbach's alpha coefficient of 0.78, further supporting the instrument's suitability for cross‐cultural research [25].

### 2.7 Statistical Analysis

Data were fed to the computer and analyzed using SPSS software package version 26.0. The Shapiro‐Wilk test showed that the variables were normally distributed (*p* < 0.05). A one‐way ANOVA test was used to compare more than two categories. Pearson coefficient was used to correlate between normally distributed quantitative variables. To predictive role of demographic characteristics, resilience, and job burnout in death anxiety, multiple linear regression analysis was performed using the simultaneously (Enter) method. The significance level was set at *p* < 0.05.

### 2.8 Ethical Considerations

The study's conduct adhered meticulously to the tenets of the revised Declaration of Helsinki. This internationally recognized ethical framework establishes guiding principles for researchers engaged in biomedical research involving human subjects. Before survey administration, participants received a concise yet comprehensive explanation of the study's purpose and were subsequently asked to provide basic demographic information. To uphold strict confidentiality standards, no personally identifiable information was collected, and informed consent was implicitly confirmed through the completion of the questionnaire—an action that simultaneously assured participants of their right to withdraw at any stage. Furthermore, the Institutional Research Ethics Committee of Fasa University of Medical Sciences in Fasa, Iran, formally approved the study (Ethical code: IR. FUMS. REC.1402.021).

## 3 Results

A total of 417 pre‐hospital emergency personnel were included in the present study. Notably, the sample population was exclusively male. The participants' mean age was 32.62 years (SD ± 4.82), ranging from 21 to 52 years. Similarly, work experience averaged 8.49 years (SD ± 6.31), ranging from 1 to 27 years. Additional demographic characteristics are presented in Table 1. The mean score for job burnout was 61.36 (SD ± 36.76), while the mean score for death anxiety was 99.5 (SD ± 94.10). These scores can be interpreted as indicative of high levels of both job burnout and death anxiety. Conversely, the mean resilience score of 96.37 (SD ± 77.06) suggests moderate resilience within the sample population (Table 2).

**Table 1:** Demographic characteristics in prehospital emergency personnel (*n* = 417).

Variable		Number	Percentage
Marital status	Single	150	36
	Married	267	64
Education level	Bachelor's degree	321	77
	Master's degree	18	4.3
	Associate's degree	78	18.7
Work experience (years)	1–9	200	48
	10–20	159	38.12
	21–27	58	13.90
Total		417	100

**Table 2:** Mean and standard deviation of resilience, job burnout, and death anxiety in prehospital emergency personnel.

Variable	Mean	Standard deviation	Minimum	Maximum
Resilience	97.06	±37.96	31	155
Job burnout	76.53	±36.61	0	132
Death anxiet**y**	10.94	±5.99	0	25

Table 3 present the findings from the correlational analyses investigating the relationships between demographic variables (age, work experience, marital status, education level, employment type) and death anxiety, job burnout, and resilience. No statistically significant correlations were observed between age, work experience, and either death anxiety, job burnout, or resilience. Table 4 presents the correlational analyses examining the relationships between death anxiety, job burnout, and resilience. The findings revealed an inverse correlation between resilience and job burnout (*r* = −0.49, *p* < 0.001) and an inverse correlation between resilience and death anxiety (*r* = −0.51 *p* < 0.001). Also, the findings revealed a statistically significant positive correlation between job burnout and death anxiety (*r* = 0.37, *p* < 0.001), indicating that individuals with higher levels of death anxiety also reported experiencing greater job burnout. Table 5 presents a linear regression analysis of significant factors affecting death anxiety. Table 5 indicates the results of multiple linear regression analysis regarding the predictive role of demographic characteristics, resilience, and job burnout in death anxiety. The results showed that age, work experience, resilience, and job burnout explained 44.4% of the variance in death anxiety (*R* ^2^ = 0.44, *p* < 0.001). In addition, work experience (*β* = 0.097, *p* = 0.043), resilience (*β* = 0. 208 *p* < 0.001), and job burnout (*β* = 0.337, *p* < 0.001) had the highest predictive impact on death anxiety.

**Table 3:** Relationship between job burnout, death anxiety, resilience and socio‐demographic data (*n* = 417).

Variable		Resilience Mean ± SD	*p* value*	Job burnout Mean ± SD	*p* value*	Death anxiety Mean ± SD	*p* value*
Age (years)	1–20	95.54 ± 38.15	0.37	76.21 ± 37.21	0.57	11.07 ± 5.96	0.41
	21–30	95.18 ± 37.67		76.71 ± 36.45		12.38 ± 5.14	
	31–52	96.38 ± 36.45		75.73 ± 35.41		10.08 ± 6.23	
		Total 97.06 ± 37.96		Total 76.53 ± 36.61		Total 10.94 ± 5.99	
Work experience (years)	1–9 years	98.50 ± 37.13	0.47	73.41 ± 35.94	0.44	10.91 ± 6.06	0.17
	10–20 years	93.67 ± 37.85		81 ± 33.77		10.55 ± 6.21	
	21–30 years	94.31 ± 40.86		88.35 ± 37.92		11.10 ± 5.94	
		Total 97.06 ± 37.96		Totall 76.53 ± 36.61		Totall 10.94 ± 5.99	
Education level	Bachelor's degree	95.54 ± 38.15	0.08	75.73 ± 37.54	0.12	11.07 ± 5.96	0.23
	Associate's degree	95.16 ± 37.67		82.92 ± 38.63		12.38 ± 5.14	
	Master's degree	96.26 ± 48.33		75.76 ± 33.28		10.08 ± 6.23	
		Total 97.06 ± 37.96		Total 75.63 ± 36.61		Total 10.94 ± 5.99	
Marital status	Single	91.68 ± 37.57	0.23	76.21 ± 37.31	0.89	10.89 ± 6.06	0.39
	Married	93.09 ± 37.91		76.71 ± 36.45		10.97 ± 5.96	
		Totall 97.06 ± 37.96		Totall 76.53 ± 36.61		Totall 10.94 ± 5.94	

* one‐way ANOVA.

**Table 4:** Relationship between job burnout, death anxiety, and resilience in the prehospital emergency personnel.

Variable		*p* value*	*r*
Resilience	Job burnout	< 0.001	0.49
Job burnout	Death anxiety	< 0.001	0.37
Resilience	Death anxiety	< 0.001	0.51

* Statistically significant at *p* < 0.05.

**Table 5:** Factors predicting missing nursing care (Adjusted *R* ^2^ = 0.44).

Variable	*B*	Beta	*t*	*p*	95% CI	
					LL	UL
**Age**	−2.457	−0.071	−0.837*	0.002*	0.010	0.402
**Gender (males)**	−5.335	−0.137	−2.925	0.374	−1.190	1.500
**Work experience (years)**	−3.438	−0.097	−2.030*	0.043*	−0.467	0.051
**Marital status**	1.025	0.036	0.575	0.565	−1.190	1.500
**Education**	−2.311	−0.143	−1.586	0.113	−1.029	0.473
**Job burnout scale**	0.172	0. 337	0.118*	< 0.001*	−0.021	0.010
**Resilience scale**	−0.663	−0.208	−4.401*	< 0.001*	−0.026	0.007
	**Adjusted *R*** ^**2**^ = **0.44**		***F*** = **1.34** *	***p*** < **0.001** *		

* Statistically significant at *p* < 0.05.

Abbreviations: B, unstandardized coefficients; Beta, standardized coefficients; LL, Lower limit; *R* ^2^, coefficient of determination; t, *t* test of significance; UL, upper limit.

## 4 Discussion

The current investigation aimed to explore the levels of resilience, death anxiety, and job burnout experienced by prehospital emergency personnel in southern Iran.

### 4.1 Death Anxiety, Resilience, and Job Burnout Levels Among Prehospital Emergency Personnel

The findings revealed concerningly high levels of death anxiety, resilience, and job burnout levels among prehospital emergency personnel. These results align with prior research by Frans et al. (2012), who documented high job burnout among prehospital emergency personnel [26]. Similarly, studies by Abdo et al. (2020) and Sporer (2021) reported elevated job burnout in this workforce [16, 27]. The high‐intensity nature of prehospital emergency care—characterized by substantial workloads, urgent decision‐making, continuous operational transitions, and the imperative for peak efficiency—necessitates strategic management and optimal resource allocation. Constrained by limited resources, unpredictable scenarios, a lack of professional recognition, and frequent interpersonal conflicts, emergency medical personnel endure considerable psychological strain. These occupational stressors markedly increase the risk of burnout among prehospital emergency professionals, underscoring the urgent need for targeted intervention strategies [28]. As a cornerstone of the healthcare system, EMS are integral in delivering immediate, life‐saving interventions. Paramedics, positioned as frontline responders in acute medical crises, operate in high‐pressure environments that demand rapid clinical judgment and involve frequent exposure to distressing or traumatic events. Consequently, these professionals are particularly vulnerable to burnout, which can detrimentally affect both their well‐being and the quality of patient care [29]. Mitigating burnout among EMS professionals necessitates a multifaceted, evidence‐based approach. Organizational initiatives should prioritize optimizing work environments, expanding access to psychological and peer support services, implementing structured shift rotations with adequate rest periods, and cultivating a workplace culture that fosters open communication, professional recognition, and emotional resilience [30]. Liu et al. (2023) reported that pre‐hospital emergency personnel experienced low levels of job burnout. This finding that contrasts with the results of the present study [31]. Also Kosydar‐Bochenek et al. reported that pre‐hospital emergency personnel experienced moderate levels of job burnout [32]. This discrepancy may be attributable to differences in measurement instruments, cultural contexts, working conditions, organizational support structures, and institutional cultures. in Iran include excessive workloads, insufficient support from superiors and the healthcare system, a dearth of rest opportunities, lack of recognition, and low remuneration. To address professional burnout among Iranian EMS personnel, concerted efforts are required at both the emergency medical unit and healthcare system levels.

In the present study findings revealed a high prevalence of death anxiety among prehospital emergency personnel. This finding is corroborated by the work of Brady (2015), who reported elevated death anxiety among prehospital emergency personnel [8]. The findings of Chegini et al. (2021) provide further corroboration. Their study documented high levels of death anxiety among prehospital emergency personnel and revealed an inverse relationship between death anxiety and job satisfaction. In essence, this suggests that as death anxiety increases, job satisfaction among this workforce correspondingly decreases [5].

The current investigation yielded a counterintuitive finding regarding the resilience of prehospital emergency personnel. While participants scored within the middle range on the employed resilience measure, extant literature paints a contrasting picture. For instance, Norouzian et al. (2022) reported low resilience levels in this population, emphasizing the potential benefits of resilience development for mental health and professional quality of life within this demanding occupational group [33]. Similarly, Fatahi et al. (2022) documented moderate resilience levels and a statistically significant positive correlation between resilience and professional quality of life, further underscoring its significance [34]. These findings suggest the need for further exploration of factors influencing resilience in prehospital emergency personnel. While the present study's results differ from previous research, the potential benefits of resilience remain well‐supported.

### 4.2 Relationship Between Death Anxiety, Resilience, and Job Burnout Levels Among Prehospital Emergency Personnel

In the present study, the findings revealed an inverse correlation between resilience and job burnout and an inverse correlation between resilience and death anxiety. Also, the findings revealed a statistically significant positive correlation between job burnout and death anxiety. As evidenced by Shojafar et al. (2014), prehospital emergency personnel often experience high levels of occupational burnout, and a direct positive relationship exists between resilience and burnout, with increasing resilience mitigating burnout [35]. Also, the current study revealed that pre‐hospital emergency personnel with higher resilience were less likely to experience death anxiety. This can be attributed to Terror Management Theory (TMT), which suggests resilient individuals are better equipped to manage death anxiety because they have internalized strong cultural worldviews and possess a robust sense of self‐worth. By reinforcing the commitment to meaningful worldviews and personal values, TMT highlights how the awareness of death can paradoxically strengthen psychological resilience, helping nurses navigate life's challenges with a greater sense of purpose and grit [36]. In the present study, the findings revealed an inverse correlation between resilience and job burnout. Similarly, Deldar et al. (2018). demonstrated a significant negative correlation between resilience and job burnout among nurses [37]. Also, the findings of Mallett, et al (1991). revealed a significant positive correlation between job burnout and death anxiety among intensive care unit nurses, thereby aligning with the results of the current study [38].

### 4.3 Relationship Between Death Anxiety, Resilience, and Job Burnout Levels and Demographic Characteristics Among Prehospital Emergency Personnel

According to the results of the present study, no statistically significant correlations were observed between age, work experience, and either death anxiety, job burnout, or resilience. Zahednezhad et al (2021). reported no significant association between nurses' demographic characteristics and their levels of resilience and job burnout, a finding that is consistent with our observations [39]. The results of the study Solaimani Moghaddam's et al. (2024), showed that there was a significant relationship between resilience and job burnout with the gender and work experience of nurses. Female nurses and nurses with more work experience experienced higher levels of resilience and lower job burnout [40]. This finding that contrasts with the results of the present study. This discrepancy may be attributable to differences in measurement instruments, institutional cultures and working conditions.

### 4.4 Predictive Role of Demographic Characteristics, Resilience, and Job Burnout in Death Anxiety

The results showed that age, work experience, resilience, and job burnout explained 44.4% of the variance in death anxiety. In addition, work experience, resilience, and job burnout had the highest predictive impact on death anxiety. In line with the findings of this study, Xie et al. found that younger nurses and those with less professional experience may lack the coping mechanisms and emotional resilience that develop over time, making them more vulnerable to the psychological impacts of frequent exposure to death and dying [41]. Du et al.'s (2024), study indicated that resilience, gender, and self‐efficacy collectively explain 43% of the variance in job burnout [42]. Moreover, Norouzi et al. (2024) identified several key determinants of death anxiety among nurses, including resilience, gender, mental health status, coping with death, and life satisfaction [43].

### 4.5 Limitation

The study utilized a convenience sampling method, which may introduce bias into the sample selection process. To prevent selection bias, sampling was conducted on different days and times, and sampling was also conducted with maximum variation. The data collection relied on self‐reported measures, the participants'responses may not have been truthful, so social desirability bias or acquiescence bias could have been present. The study employed a cross‐sectional design, which captured data at a single point in time. This design does not allow for examining causal relationships between variables or changes over time. The study calls for further research to explore the relationship between resilience, job burnout, and death anxiety among prehospital emergency personnel, with longitudinal studies potentially providing valuable insights into the effectiveness of interventions aimed at enhancing resilience and reducing death anxiety and job burnout.

### 4.6 Strengths

This study distinguishes itself by undertaking the inaugural exploration of the interrelatedness between death anxiety, resilience, and occupational burnout within the population of prehospital emergency personnel in southern Iran. This novel contribution to the existing body of knowledge enhances our understanding of the mental health landscape faced by this critical workforce.

### 4.7 Implications in Clinical Practice

Based on the results of the present study, pre‐hospital emergency personnel experience high levels of death anxiety and job burnout. Given the inherently stressful nature of pre‐hospital emergency work, where personnel consistently face complex and unpredictable situations, attention to their psychological safety and psychological capabilities is of considerable importance. Senior management of pre‐hospital emergency systems should prioritize continuous and systematic assessment of mental health and job burnout among prehospital emergency personnel. They should identify occupational factors affecting mental health and job burnout, and implement appropriate measures and planning accordingly. In this context, interventions such as psychological counseling and conducting continuing education programs to enhance mental health, resilience capacity, emotional intelligence, and stress management could prove effective. Additionally, measures aimed at improving working conditions and motivational factors for personnel may be beneficial.

## 5 Conclusion

The current investigation yielded concerning findings regarding the mental health of prehospital emergency personnel. Participants reported experiencing high levels of death anxiety and job burnout, while resilience scores fell within the moderate range. A thorough understanding of the factors contributing to burnout among paramedics is essential for designing targeted and effective intervention strategies. Efforts should emphasize comprehensive stress management programs, robust institutional support systems, and holistic well‐being initiatives. Moreover, recognizing gender‐specific variations in burnout experiences is vital for tailoring interventions to address the unique challenges encountered by different groups. The implementation of proactive psychological support frameworks, together with the optimization of workplace conditions, can play a pivotal role in enhancing paramedics'overall well‐being and professional efficacy. Furthermore, integrating stress management training into medical education curricula can equip paramedics with essential coping strategies, enabling them to navigate occupational stress more effectively. By fostering resilience and reinforcing psychological preparedness, such measures not only mitigate the risk of burnout but also elevate the quality of emergency medical care provided.

## Author Contributions

**Ali Ghahramanipirsalami:** investigation, methodology, data curation, conceptualization. **Farzaneh Modaresi:** conceptualization, methodology, investigation. **Parisa Sabetsarvestani:** investigation, data curation, writing – original draft. **Azizallah Dehghan:** conceptualization, methodology, software. **Saeed Abedi:** data curation, investigation. **Mostafa Bijani:** conceptualization, investigation, writing – original draft, writing – review and editing, supervision.

## Ethics Statement

Written informed consent to participate in the current investigation was obtained from all participants. The study's conduct adhered meticulously to the tenets of the revised Declaration of Helsinki. This internationally recognized ethical framework establishes a set of guiding principles for researchers engaged in biomedical research involving human subjects. Participants were explicitly assured of both anonymity and the strict confidentiality of their data. Furthermore, the Institutional Research Ethics Committee of Fasa University of Medical Sciences, situated in Fasa, Iran, formally approved the study (Ethical code: IR.FUMS.REC.1402.021).

## Conflicts of Interest

The authors declare no conflicts of interest.

## Transparency Statement

The lead author Mostafa Bijani affirms that this manuscript is an honest, accurate, and transparent account of the study being reported; that no important aspects of the study have been omitted; and that any discrepancies from the study as planned (and, if relevant, registered) have been explained.

## Data Availability

The data that support the findings of this study are available from the corresponding author upon reasonable request.
